# P66SHC deletion improves fertility and progeric phenotype of late‐generation TERC‐deficient mice but not their short lifespan

**DOI:** 10.1111/acel.12448

**Published:** 2016-03-10

**Authors:** Marco Giorgio, Massimo Stendardo, Enrica Migliaccio, Pier Giuseppe Pelicci

**Affiliations:** ^1^ Experimental Oncology DepartmentEuropean Institute of OncologyVia Ripamonti 43520141MilanItaly

**Keywords:** fertility, lifespan, oxidative DNA damage, telo‐meres

## Abstract

Oxidative stress and telomere attrition are considered the driving factors of aging. As oxidative damage to telomeric DNA favors the erosion of chromosome ends and, in turn, telomere shortening increases the sensitivity to pro‐oxidants, these two factors may trigger a detrimental vicious cycle. To check whether limiting oxidative stress slows down telomere shortening and related progeria, we have investigated the effect of p66SHC deletion, which has been shown to reduce oxidative stress and mitochondrial apoptosis, on late‐generation TERC (telomerase RNA component)‐deficient mice having short telomeres and reduced lifespan. Double mutant (TERC ^−/−^ p66SHC ^−/−^) mice were generated, and their telomere length, fertility, and lifespan investigated in different generations. Results revealed that p66SHC deletion partially rescues sterility and weight loss, as well as organ atrophy, of TERC‐deficient mice, but not their short lifespan and telomere erosion.

Therefore, our data suggest that p66SHC‐mediated oxidative stress and telomere shortening synergize in some tissues (including testes) to accelerate aging; however, early mortality of late‐generation mice seems to be independent of any link between p66SHC‐mediated oxidative stress and telomere attrition.

## Introduction

Telomeres are conserved repetitive DNA sequences at the ends of chromatids that protect from chromosomal rearrangements and loss of genetic information (Blackburn *et al*., 2006). Shortening of telomeres occurs in somatic cells during aging (Daniali *et al*., 2013) due to DNA damage and incomplete end processing (von Zglinicki *et al*., 2000). Above a threshold, telomere erosion results in the arrest of cellular proliferation and the dysfunction of renewable tissues. For this reason, telomerase activity and recombination‐based processes that maintain telomere length are critical for tissue homeostasis (Sahin & Depinho, 2010). In fact, mutations in either the gene encoding the telomerase catalytic subunit (telomerase reverse transcriptase, TERT), or the telomerase RNA gene (TERC) are found in patients with dyskeratosis congenita who have short telomeres and show accelerated aging and reduced lifespan (Kirwan & Dokal, 2009). Consistently, late‐generation (G3 and beyond) TERT (Rudolph *et al*., 1999) or TERC‐deficient mice (TERT^−/−^ and TERC^−/−^, respectively) are short living and show reduced fertility, early alopecia, kyphosis, anemia, and lymphopenia (Wong *et al*., 2003). Interestingly, TERC^−/−^ mice present altered mitochondrial functions, including increased production of reactive oxygen species (ROS) from the electron transfer chain (ETC) (Passos *et al*., 2010; Sahin *et al*., 2011). Thereby, telomeres have been suggested to play a role in controlling mitochondrial ROS accumulation and oxidative stress during aging (Sahin & Depinho, 2010).

As telomere DNA is particularly sensitive to oxidative damage (Oikawa & Kawanishi, 1999) and single‐strand breaks or other stress‐induced lesions at telomeres are inefficiently repaired (Petersen *et al*., 1998), oxidative stress is considered to accelerate telomere erosion (von Zglinicki, 2002; Houben *et al*., 2008). Consistently, mice expressing a variant of the mitochondrial uncoupling protein 2 that increases ROS production by ETC, and therefore, oxidative stress shows short telomeres (Salpea *et al*., 2010). Moreover, it has been shown that treating mouse embryos with chemicals able to induce mitochondrial dysfunction and ROS accumulation, led to telomere loss and chromosomal instability, which was prevented by a concomitant treatment with *N*‐acetylcysteine, a compound that improves ROS scavenging (Liu *et al*., 2002). However, other mutations in genes involved in ROS metabolism, such as SOD2 haploinsufficiency, do not cooperate with telomere dysfunction (Guachalla *et al*., 2009), and the treatment with *N*‐acetylcysteine does not rescue progeric phenotypes of late‐generation TERT‐deficient mice (Sahin *et al*., 2011).

P66SHC protein is the largest isoform encoded by the ShcA locus, typical of vertebrates, and it sustains intracellular levels of ROS (Giorgio *et al*., 2005 Gertz & Steegborn, 2010) and regulates redox signaling pathways (Frijhoff *et al*., 2014), mitochondrial apoptosis (Migliaccio *et al*., 2006), and aging (Trinei *et al*., 2009). P66SHC null mice (p66SHC^−/−^) develop normally and resulted to be protected from aging‐associated diseases, such as atherosclerosis (Martin‐Padura *et al*., 2008), diabetes compliances (Menini *et al*., 2007), onset (Tomilov *et al*., 2011), cognitive decline (Berry *et al*., 2007), and neurodegeneration (Savino *et al*., 2013). Moreover, p66SHC null cells were shown to be resistant to apoptosis induced by a variety of different signals, including hydrogen peroxide, UV, staurosporine, taxol, growth factor deprivation, calcium ionophore, osmotic shock, and CD3–CD4 cross‐linking (as reviewed in Migliaccio *et al*., 2006). Similarly, different tissues of p66SHC^−/−^ mice were found to be resistant to apoptosis induced by paraquat (Migliaccio *et al*., 1999), hypercholesterolemia (Napoli *et al*., 2003), hyperglycemia (Menini *et al*., 2007), immunotoxicity (Su *et al*., 2012), and ischemia/reperfusion injury (Carpi *et al*., 2009). At mechanistic level, substantial evidence indicates that p66SHC regulates redox balance and mitochondrial apoptosis by suppressing ROS scavenging and increasing ROS production from plasma membrane oxidases and mitochondria where, in particular, p66SHC favors H_2_O_2_ production by ETC (Gertz & Steegborn, 2010; Trinei *et al*., 2013). Accordingly, p66SHC^−/−^ tissues showed a reduced level of oxidative damage to nuclear and mitochondrial DNA (Trinei *et al*., 2002), lipids (Napoli *et al*., 2003), carbohydrates (Menini *et al*., 2007), and proteins (Carpi *et al*., 2009), which can be either unspecifically or specifically (e.g., redox‐regulated phosphatases; Frijhoff *et al*., 2014) damaged by oxidative stress.

To investigate the link between endogenously generated oxidative stress, telomeres, and aging, we have investigated the effect of p66SHC deletion on late‐generation TERC‐deficient mice, by generating and studying double mutants TERC^−/−^ P66SHC^−/−^ mice.

## Results

### The deletion of p66SHC improves fertility of late‐generation TERC^−/−^ mice

At first, we backcrossed TERC^+/−^ male with C57Bl6/J wild‐type (WT) female mice to generate TERC^+/−^ mice in pure C57Bl/6 background (100% C57BL/6 according to the Marker‐Assisted Accelerated Backcrossing, MAX‐BAX^®^, analysis). These mice were bred together to generate TERC^−/−^ (p66SHC^+/+^) mice that were bred successfully, by crossing mice of the same generation, up to the fifth generation (G5), as already reported (Rudolph *et al*., 1999).

**Figure 1:**
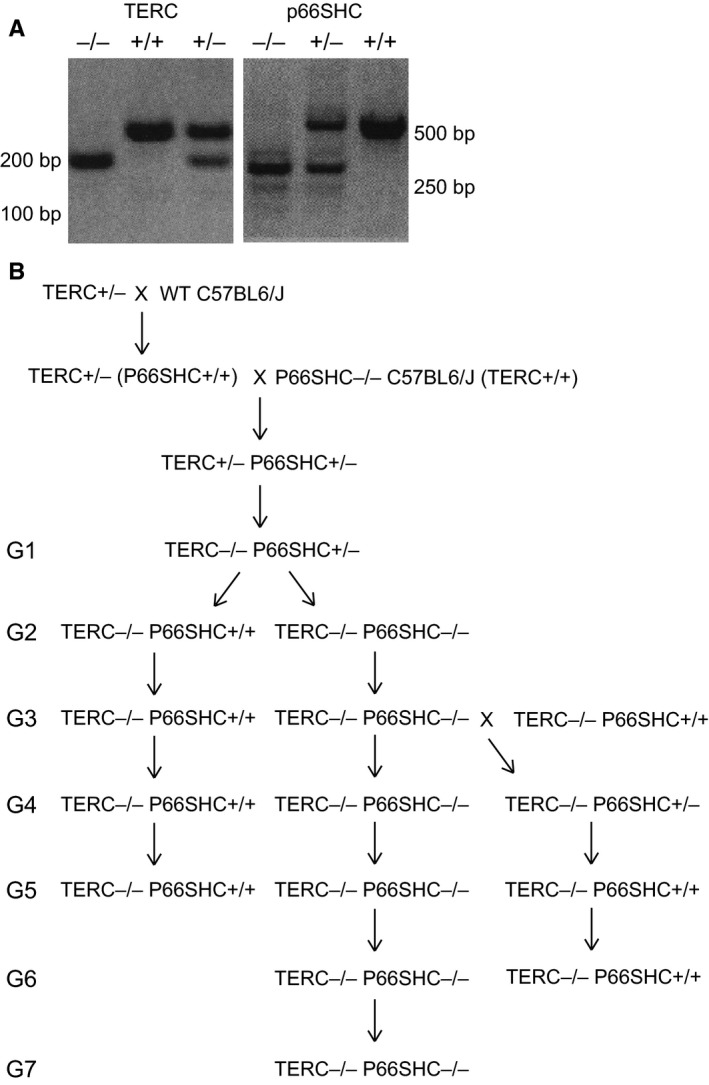
Genealogy of TERC ^−/−^ p66SHC ^+/+^ and TERC ^−/−^ p66SHC ^−/−^ mice. (A) PCR genotype analysis for TERC (left panel) and p66SHC mutation (right panel). (B) Scheme of the crosses performed to generate the different generations of TERC ^−/−^ p66SHC ^+/+^ and TERC ^−/−^ p66SHC ^−/−^ mice.

**Figure 2:**
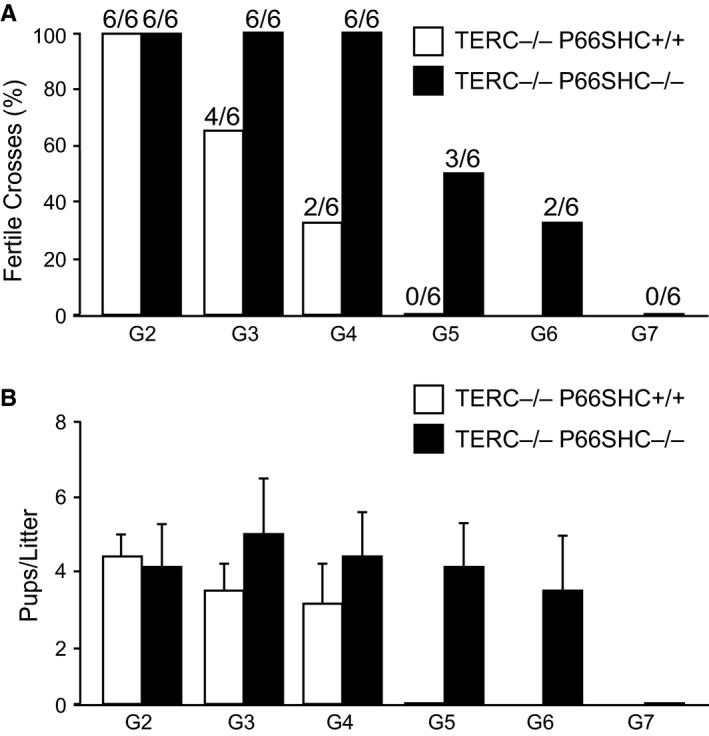
Fertility of different generations of TERC ^−/−^ p66SHC ^+/+^ and TERC ^−/−^ p66SHC ^−/−^ mice. (A) Percentage of productive crosses along generations (*n* = 6 couples per genotype per generation). (B) Average number ±SD of pups per litter along generations.

Then, we crossed C57Bl/6J TERC^+/−^ female with pure C57Bl/6J p66SHC^−/−^ male mice and selected TERC^+/−^ p66SHC^+/−^ double heterozygous mice among the resulting litters. These mice were then crossed together and litters genotyped for TERC and p66SHC mutations (Fig. 1A). The resulting TERC^−/−^ p66SHC^+/−^ mice were selected as the first generation (G1) of TERC null mice and were bred together to segregate into three genotypes: p66SHC^−/−^, p66SHC^+/−^, and p66SHC^+/+^ (Table 1). In this way, we obtained G2 TERC^−/−^ p66SHC^+/+^ and G2 TERC^−/−^ p66SHC^−/−^ mice that were propagated as distinct colonies by breeding mice from the same generations (Fig. 1B). As observed for the other colonies of TERC^−/−^ mice, at G5 TERC^−/−^ p66SHC^+/+^ crosses were not fertile, as well as the crosses of males (or females) G5 TERC^−/−^ p66SHC^+/+^ with WT female (or male) mice. On the opposite, half of the crosses of G5 and also approximately a third of the G6 TERC^−/−^ p66SHC^−/−^ mice were fertile, and thus, it was possible to generate G7 TERC^−/−^ p66SHC^−/−^ mice (Fig. 2A).

**Table 1:** Frequency of p66SHC^+/+^, p66SHC^+/−^, and p66SHC^−/−^ genotypes in the progeny obtained crossing G1 TERC^−/−^ p66SHC^+/−^ mice

GENOTYPE	Number	Males	Females	Expected (%)	Observed (%)
TERC^−/−^P66SHC^+/−^	35	18	17	50	57.4
TERC^−/−^P66SHC^+/+^	10	6	4	25	16.4
TERC^−/−^P66SHC^−/−^	16	8	8	25	26.2
Total	61	32	29	100	100

The average number of pups per dam was comparable between G2 TERC^−/−^ p66SHC^+/+^ and G2 TERC^−/−^ p66SHC^−/−^ mice, as well as between WT and p66SHC^−/−^ animals (Giorgio *et al*., 2012). At G3, TERC^−/−^ p66SHC^+/+^, but not TERC^−/−^ p66SHC^−/−^, mice showed a significant fertility reduction. Only, at G5, we observed a decrease in litter size of TERC^−/−^ p66SHC^−/−^ mice (Fig. 2B).

Furthermore, we crossed G3 TERC^−/−^ p66SHC^+/+^ females with TERC^−/−^ p66SHC^−/−^ male mice to produce G4 TERC^−/−^ p66SHC^+/−^ mice. By crossing these mice, we could segregate other G5 TERC^−/−^ p66SHC^+/+^ animals. Interestingly, one of four crosses of these G5 TERC^−/−^ p66SHC^+/+^ was fertile and two males of four of the resulting G6 TERC^−/−^ p66SHC^+/+^ litter were able to generate offspring when crossed with WT females.

These data clearly suggest that p66SHC deletion improves fertility of late‐generation TERC^−/−^ mice.

### The deletion of p66SHC prevents DNA oxidative damage but not telomere shortening in TERC^−/−^ mice

**Figure 3:**
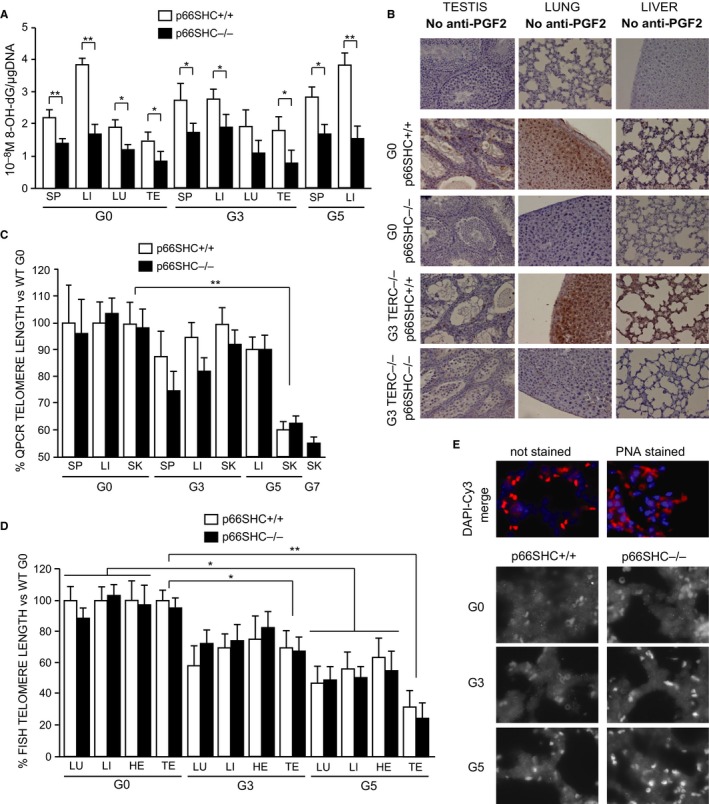
Oxidative stress and telomere length in TERC ^−/−^ p66SHC ^+/+^ and TERC ^−/−^ p66SHC ^−/−^ mice. (A) Levels of 8‐OH‐dG per μg of DNA in TERC ^−/−^ p66SHC ^+/+^ (white bars) and TERC ^−/−^ p66SHC ^−/−^ (black bars) spleen (SP), liver (LI), lung (LU), and testis (TE) measured by competitive enzyme immunoassay. Average values ±SD from *n* = 5 individuals for the G0 group and *n* = 3 individuals for the G3 and G5 groups are reported: * for *P *< 0.05 and ** for *P* < 0.01. (B) 8‐isoprostane IHC representative images of testis, lung, and liver from G0 and G3 TERC ^−/−^ p66SHC ^+/+^ and TERC ^−/−^ p66SHC ^−/−^ mice. Level of background signal is shown in the first three unstained slices. (C and D) Telomere length (expressed as a percentage versus the telomere length of C57Bl/6J WT mice) in spleen (SP), liver (LI), lung (LU), heart (HE), testis (TE) and skin (SK) from TERC ^−/−^ p66SHC ^+/+^ and TERC ^−/−^ p66SHC ^−/−^ mice of different generation as evaluated by qPCR (C) where the average values ±SD from *n* = 3 individuals per group, measured in quadruplicates, are reported or Q‐FISH (D) where the average values ±SD from *n* = 3 individuals per group, each sample evaluated from two different sections for a total of around 40 nuclei, are reported. * for *P* < 0.05, ** for *P* < 0.01. (E) Representative FISH images (Cy3 alone grayscale and DAPI‐Cy3 merged color) obtained from the lung of different generation TERC ^−/−^ p66SHC ^+/+^ and TERC ^−/−^ p66SHC ^−/−^ mice.

Eight‐hydroxy‐2‐deoxy guanosine (8‐OH‐dG) and isoprostanes are recurrent oxidized DNA base and fatty acids products, respectively, that marks oxidative stress to DNA and lipids and were found reduced in p66SHC^−/−^ mice (Trinei *et al*., 2002; Napoli *et al*., 2003; Lunghi *et al*., 2015). We have measured the content of 8‐OH‐dG in the genomic DNA extracted from liver, spleen, lung, and testis of G0 and G3; and liver and spleen of G5 TERC^−/−^ p66SHC^+/+^ or TERC^−/−^ p66SHC^−/−^ 6‐month‐old mice using a competitive ELISA assay with specific anti‐8‐OH‐dG antibody and of 8‐iso‐PGF2α (8‐isoprostane) by immunohistochemistry analysis of tissue sections from testis, lung, and liver of G0 and G3, TERC^−/−^ p66SHC^+/+^, and TERC^−/−^ p66SHC^−/−^ mice. Results showed that the deletion of p66SHC reduced the amount of 8‐OH‐dG (Fig. 3A) and of 8‐isoprostane (Fig. 3B) in TERC^−/−^ genetic background as well.

To determine telomere length, we performed qPCR analysis of telomeric sequences in genomic DNA extracted from skin, spleen, and liver of 6‐month‐old G3–G5 TERC^−/−^ p66SHC^+/+^, G3‐G7 TERC^−/−^ p66SHC^−/−^, and WT mice (Fig. 3C), and quantitative fluorescent *in situ* hybridization (Q‐FISH) analysis on tissue sections from liver, lung, heart, and testis of 6‐month‐old G0, G3, and G5 TERC^−/−^ p66SHC^+/+^ or TERC^−/−^ p66SHC^−/−^ mice (Figs 3 D and E and S1 in Supporting information). As expected, we observed a progressive telomere shortening in successive generations of TERC^−/−^ mice, particularly in the skin by the qPCR analysis and more evident in all the tissues by Q‐FISH. However, the TERC^−/−^ p66SHC^+/+^ and TERC^−/−^p66SHC^−/−^ showed a similar telomere decline, suggesting that p66SHC does not contribute to telomere erosion.

### The deletion of p66SHC prevents weight loss and organ shrinkage in late‐generation TERC^−/−^ mice

**Figure 4:**
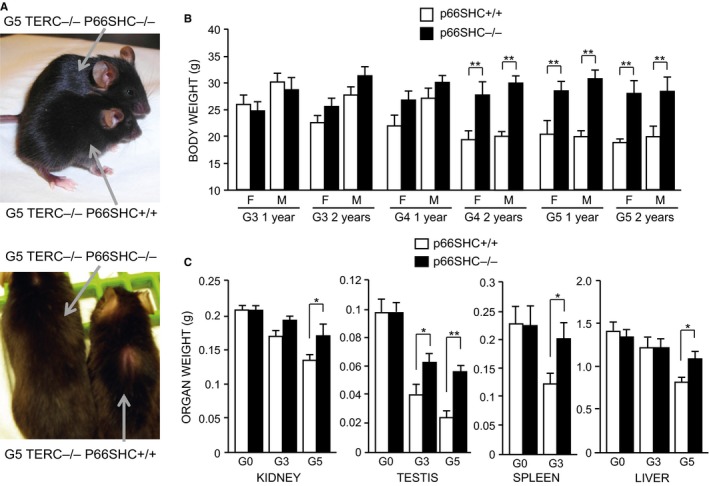
Appearance and weight of TERC ^−/−^ p66SHC ^+/+^ and TERC ^−/−^ p66SHC ^−/−^ mice. (A) Representative images of 3 months old G5 TERC ^−/−^ p66SHC ^+/+^ and TERC ^−/−^ p66SHC ^−/−^ mice. (B) Mean body weight of TERC ^−/−^ p66SHC ^+/+^ and TERC ^−/−^ p66SHC ^−/−^ mice at 1 and 2 years of age (F, female; M, male), *for p < 0.05 ** for *P* < 0.01. (C) Kidney, testis, spleen, and liver weight of G0–G5 TERC ^−/−^ p66SHC ^+/+^ and TERC ^−/−^ p66SHC ^−/−^ mice.

**Figure 5:**
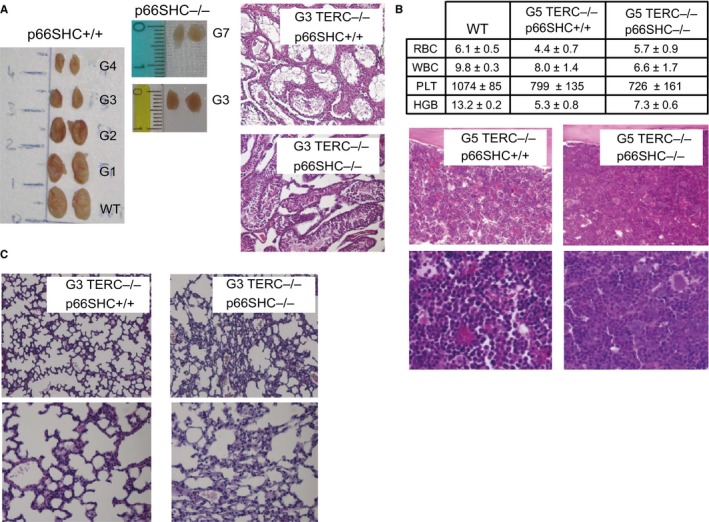
Phenotypic characterization of TERC ^−/−^ p66SHC ^+/+^ and TERC ^−/−^ p66SHC ^−/−^ mice. (A) Representative images of morphology (left) and histology (right) of testes from TERC ^−/−^ p66SHC ^+/+^ and TERC ^−/−^ p66SHC ^−/−^ mice. (B) Blood counts (upper table: WBC 10^3^/μL, RBC 10^6^/μL, PLT 10^3^/μL, HGB mg dL^−1^; *n* = 6 mice per group) and bone marrow histology of TERC ^−/−^ p66SHC ^+/+^ and TERC ^−/−^ p66SHC ^−/−^ mice. (C) Histological examination of lung tissues from G5 TERC ^−/−^ p66SHC ^+/+^ and TERC ^−/−^ p66SHC ^−/−^ mice.

Along with reduced fertility, starting from G3, C57Bl6/J TERC^−/−^ mice showed signs of premature aging as already reported (Wong *et al*., 2003; Siegl‐Cachedenier *et al*., 2007). At first sight, young adult (2–3 months old) TERC^−/−^ p66SHC^+/+^ mice exhibited alopecia on the back and kyphosis and were of smaller size compared to G3 TERC^−/−^ p66SHC^−/−^ mice that appeared bigger and healthier (Fig. 4A). At G5, TERC^−/−^ p66SHC^+/+^ mice were significantly smaller compared to age‐matched TERC^−/−^ p66SHC^−/−^ mice (Fig. 4B). We observed a reduction in the size and weight (Fig. 4C) of a number of organs including liver, spleen, kidneys, and testis, in late‐generation TERC^−/−^ p66SHC^+/+^ mice; in particular, starting from G3 these mice presented testis atrophy that, however, was not observed in TERC^−/−^ p66SHC^−/−^ mice (Fig. 5A). Notably, the frequency of apoptotic cells as evaluated by IHC analysis with anti‐activated caspase‐3 antibody appeared significantly reduced in the testis from p66SHC^−/−^ background (Fig. S2). Aplastic anemia has been also associated to telomere shortening in both humans and mouse models (Siegl‐Cachedenier *et al*., 2007); however, blot tests and bone marrow examination did not evidence severe aplastic anemia either in TERC^−/−^ p66SHC^+/+^ or TERC^−/−^ p66SHC^−/−^ mice (Fig. 5B). No clear signs of muscle degeneration but rare loss of the striations, vacuolization, and infiltrations in the G3 TERC^−/−^ p66SHC^+/+^, as shown in the Fig. S3, were observed. Abnormal glycaemia was not revealed in all generations as well. Lung emphysema was observed in late‐generation TERC^−/−^ regardless the p66SHC genotype, whereas lung fibrosis was markedly evident in p66SHC^−/−^ mice (Fig. 5C).

### P66SHC does not affect the lifespan of late‐generation TERC^−/−^ mice

**Figure 6:**
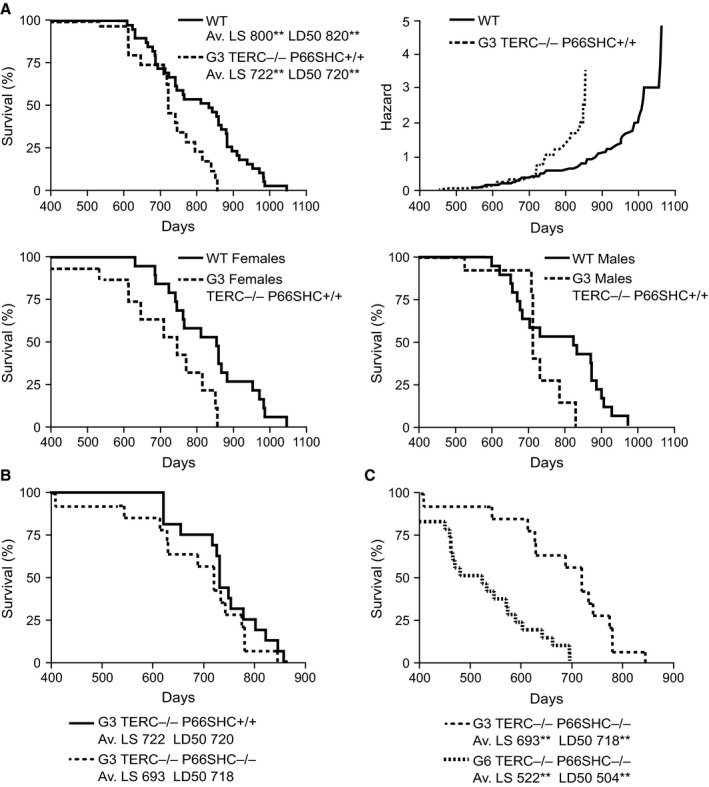
Survival curves of WT, TERC ^−/−^ p66SHC ^+/+^, and TERC ^−/−^ p66SHC ^−/−^ mice. (A) Left upper graph, WT (*n* = 40) and G3 TERC ^−/−^ p66SHC ^+/+^ (*n* = 18) male and female mice (Av. LS, average lifespan; LD50, median life time; **P < 0.01 for the effect of TERC genotype); right upper graph, cumulative hazard function for WT and G3 TERC ^−/−^ p66SHC ^+/+^ male and female mice only; left lower graph, WT (*n* = 20) and G3 TERC ^−/−^ p66SHC ^+/+^ (*n* = 10) female mice; right lower graph, WT (*n* = 20) and G3 TERC ^−/−^ p66SHC ^+/+^ (*n* = 8) male mice only. (B) G3 TERC ^−/−^ p66SHC ^+/+^ (*n* = 18) and G3 TERC ^−/−^ p66SHC ^−/−^ (*n* = 14) male and female mice. (C) G3 TERC ^−/−^ p66SHC ^−/−^ (*n* = 14) and G6 TERC ^−/−^ p66SHC ^−/−^ (*n* = 22) male and female mice (Av. LS and LD50 **P < 0.01 for the effect of generation).

G3 C57Bl/6 TERC^−/−^ mice were shown to be short living (Rudolph *et al*., 1999). The comparison of survival curves of WT and G3 TERC^−/−^ p66SHC^+/+^ mice confirmed this observation (Fig. 6A). In particular, mortality risk (Fig. 6A, right upper panel) was comparable for the first 2 years, whereas starting from the third year it increases much more for TERC^−/−^ mice than for WT; G3 TERC^−/−^ p66SHC^+/+^ males showed the shortest survival.

The survival rate of G3 TERC^−/−^ p66SHC^−/−^ was identical to that of G3 TERC^−/−^p66SHC^+/+^ mice (Fig. 6B). They showed the same reduction in survival compared to the WT and p66SHC^−/−^ (TERC^+/+^) mice. We could also determine the lifespan of G6 TERC^−/−^ p66SHC^−/−^ mice, revealing a further shortening of lifespan with respect to the G3 (Fig. 6C). Necroscopic examination of spontaneously dead G3 and G6 mice revealed the presence of visible tumor masses only in few cases (12 of 100 mice), equally distributed with respect to p66SHC mutation, rare enlargement of bladder and fecal prolapse, no splenomegaly or enlarged lymph nodes, and no hemorrhagic tissues or ascites.

In particular, the average body weight of G6 TERC^−/−^ p66SHC^−/−^ mice was 18.5 ± 0.4 g at 3 months of age and 23.1 ± 1.8 g at 1 year of age. They showed tremor and lethargy because 6–8 months of age and frequently hair loss around the nose and the limb, no abnormalities in the blood formula but a mild anemia, normal glycaemia and lipidic profile, and small lungs and thymus at death.

## Discussion

Consecutive crosses of TERC^−/−^ mice originate individuals with insufficient telomere length, organ dysfunctions, low fertility, and short lifespan (Wong *et al*., 2003 and Fig. 6A).

Oxidative stress shortens telomeres (von Zglinicki, 2002), and the deletion of p66SHC reduces oxidative stress (Trinei *et al*., 2002 and Fig. 3A and B). However, in the double mutants (TERC^−/−^ p66SHC^−/−^) mice, we do not observe differences of telomere length with respect to the TERC^−/−^ p66SHC^+/+^ (Fig. 3 C and D). This result indicates that the effect of reducing oxidative damage, by deleting p66SHC, on telomere shortening is less important with respect to other reasons of telomere erosion such as the incomplete replication of chromosomal ends.

The function of p66SHC appears instead relevant for the downstream consequences of dysfunctional chromosome ends, as p66SHC deletion improved fertility and reduce weight and shrinkage of different organs including testis of late‐generation TERC^−/−^ mice (Fig. 4). Short telomeres of late‐generation TERC^−/−^ mice induce senescence and apoptosis (Wong *et al*., 2003) that determine the detrimental effects of telomere erosion ultimately. P66SHC triggers mitochondrial apoptosis upon a variety of stresses (Migliaccio *et al*., 2006). At mechanistic level, p66SHC is plausible to mediate cell loss following telomere erosion rather than having a role in telomere shortening.

Regardless of the recovered fertility, weight, and healthier appearance, the p66SHC deletion does not improve longevity of G3 TERC^−/−^ mice (Fig. 6B). Indeed, lung emphysema and fibrosis are frequent in both G3 and later generations of TERC^−/−^ independently by the p66SHC deletion, although we could not establish that lung dysfunction is the cause of death of these mice. Histiocytic sarcoma was reported to be the most frequent tumor lesion in C57Bl/6 mice, including p66SHC^−/−^ (Ramsey *et al*., 2014) and TERC^−/−^ (Khoo *et al*., 2007) models. In any case, the early mortality of TERC^−/−^ mice is not determined by p66SHC suggesting that oxidative stress and telomere attrition do not cooperate to determine lifespan. In this view, the unification of mitochondrial (free radicals) and nuclear (telomere) theories of aging (Sahin & DePinho, 2012) appears uncertain since the peculiar reasons of death of laboratory C57BL/6 mice.

From an evolutionary point of view, short telomeres and repressed telomerase (as in human somatic tissues) have been suggested to co‐evolve with homeothermy and replicative aging in mammals (Gomes *et al*., 2011), while p66SHC with the metabolic adaptation to harsh energetic environments (i.e., food deprivation and cold temperature) (Giorgio *et al*., 2012). In this view, the relative healthier aging of TERC^−/−^ p66SHC^−/−^ compared to TERC^−/−^ p66SHC^+/+^ mice suggests that the cost to pay for having a plastic metabolism (as ensured by p66SHC) is to amplify the negative effect of telomere erosion on body size and fertility.

Finally, results from the study of p66SHC/TERC support the notion that genetic factors control the response to telomere erosion of specific organs. Thus, the predictive value of telomere length on organ aging depends on genetic background. Then, p66SHC expression and activity is induced in several tissues by obesogenic diets (Giorgio *et al*., 2012) or hyperglycemia (Albiero *et al*., 2014) that are known to affect telomere length as well (Laimer *et al*., 2015). The interaction of aging and metabolic pathways, including p66SHC, SIRT1, or AMPK/mTOR/S6K (Fadini *et al*., 2011), with telomere erosion in the presence of diabetes or obesity is a promising field to reveal how metabolic disorders impact on aging.

## Experimental procedures

### Mice

P66SHC mice were generated in our laboratory, at the European Institute of Oncology (Milan, Italy); TERC^+/−^ mice (Lee *et al*., 2001) originated from the laboratory of Ronald DePinho (Dana‐Farber Cancer Institute, 450 Brookline Ave. Boston, MA, USA), and WT C57Bl6/j mice were purchased from Charles River Laboratories, Italy. All mice were housed at the specific‐pathogen‐free FELASA‐certified mouse facility in a temperature‐controlled room (temperature 21 ± 1 °C, relative humidity 60 ± 10%) under a 12‐h light/12‐h dark cycle (lights on from 7:00 a.m. to 7:00 p.m.) and with ad libitum food availability (2018S Teklad Global 18% Protein Rodent Diet; Harlan Laboratories Lesmo, Italy) and drinking water (autoclaved tap water). Proper group housing (four animals per cage) was chosen to improve animal welfare. Home cages were Plexiglas boxes (42 × 27 × 14 cm) with sawdust as bedding.

Experiments have been carried out in accordance with institutional guidelines and the Italian law, which enforces EU directives regarding the protection of animals used for experimental and other scientific purpose.

### TERC and p66SHC genotyping

The primers and reaction conditions used for PCR genotyping were as follows: for TERC mouse mutants, 5′ GGG GCT GCT AAA GCG CAT 3′; 5′ TTC TGA CCA CCA CCA ACT TCA AT 3′; 5′ CTA AGC CGG CAC TCC TTA CAA G 3′; and reactions: 95 °C 15 min, 94 °C 1 min, −55 °C 1 min, −72 °C 1 min (35 cycles), 72 °C 7 min. For p66SHC mouse mutants: 5′ CTC GTG TGG GCT TAT TGA CAA AG 3′; 5′ CCT CCC CAG GTC ATC TGT TAT CC 3′; 5′ GGG TGG AGA GGC TTT TTG CTT C 3′; and reactions: 95 °C 15 min, 94 °C 1 min, −64 °C 1 min, −72 °C 1 min (35 cycles), 72 °C 7 min.

### Telomere length qPCR and Q‐FISH assays

All buffers were purged with nitrogen and supplemented with 50 μm phenyl‐tert‐butyl nitrone (Sigma‐Aldrich Milan, Italy Srl) to prevent oxidation and minimize oxidative damage to DNA, which may alter the efficiency of the PCR if abasic sites are generated. Genomic DNA was isolated from mouse tissues using the silica‐gel‐membrane‐based DNeasy Tissue Kit (QIAGEN, Italy Srl Milan, Italy), according to the manufacturer's protocol with minor modifications. To minimize abasic site generation, the initial high temperature lysis with proteinase K was replaced by 6 hours incubation at 37 °C. Following the elution of purified DNA, 1 mm DTT (dithiothreitol) was added to the DNA samples, which were stored at −80 °C until use. DNA was quantified in triplicate using a NanoDrop spectrophotometer (Thermo Fisher Scientific Inc. Waltham, MA USA). Each PCR (20 μL) was performed as follows: 20 ng DNA, 1 × SYBR^®^ Green master mix (Life Technologies‐Applied Biosystems Italia Monza, Italy), 100 nm telomere forward primer (CGG TTT GTT TGG GTT TGG GTT TGG GTT TGG GTT TGG GTT), and 100 nm telomere reverse primer (GGC TTG CCT TAC CCT TAC CCT TAC CCT TAC CCT TAC CCT) (O'Callaghan *et al*., 2008). Results are from the measure of three individuals per group performed in quadruplicates.

Q‐FISH was performed on 5 μm deparaffinized sections treated with 0.1 mg mL^−1^ RNA A solution and then with 0.5 mg mL^−1^ proteinase K solution for 20 min each time at 37 ^o^C. Slices were then prewarmed at 85 ^o^C for 5 min and incubated with 200 nm solution of Cy3 conjugated PNA TelC probe (PNA Bio) in 60% formamide, 10 mm Tris–HCl pH 7.5, and 0.2 μg mL^−1^ salmon sperm first for 10 min at 85 ^o^C and then for 2 h at RT. Then, the probe was washes twice with 2XSSC, 0.1% Tween‐20 solution for 10 min at 58 ^o^C. DAPI staining was used for the staining of the nucleus.

Images were collected through an Olympus BX51 upright fluorescent microscope equipped with a cool snap EZ CCD camera (Photometrics Tucson, AZ, USA) and processed by Metamorph (Molecular Device Sunnyvale, CA, USA) and ImageJ software. The Cy3 fluorescence intensity of at least 40 nuclei observed in at least two different slices per experimental group was compared (Meeker *et al*., 2002).

### 8‐OH‐dG and 8‐isoprostane analysis

8‐OH‐dG was measured using the commercially available immunoassay kit (Abcam Cambridge, UK) on genomic DNA. One microgram of total genomic DNA was denatured 5 min at 95 °C and digested with nuclease P1 (Sigma‐Aldrich Srl), then dephosphorylated by incubation with Antarctic phosphatase (New England Biolabs; EuroClone S.P.A. Pero, Italy) and loaded in duplicate on the anti‐8‐OH‐dG antibody‐coated plate. The amount of 8‐OH‐dG in the samples was quantified by measuring competition with labeled 8‐OH‐dG for antibody binding.

8‐isoprostane was detected by immunohistochemistry on deparaffinized 10 μm sections upon unmasking treatment for 45 min at 90 ^o^C with sodium citrate, 10 min of peroxide blocking at RT and decoration with anti‐8‐epi‐PGF2α (Oxford Biomedical Research Rochester Hills, MI, USA) diluted 1:500 as primary antibody, and then with biotinylated anti‐goat and VECTASTAIN ABC Kit (Vector laboratories Burlingame, CA, USA) followed by minimal counterstaining with hematoxylin.

### Tissue analysis

Blood formula was obtained using the Haematological Analyzer Act 5 Diff (Beckman Dickinson Franklin Lakes, NJ, USA). Histological analysis was performed on fresh tissues that were rapidly washed in PBS and incubated in 4% formaldehyde‐buffered solution for 16 h at room temperature. Fixed samples were then dehydrated by increasing concentrations of ethanol and included in paraffin using the Leica ASP300 tissue processor (Leica Biosystems – Leica Microsystems Srl Milan, Italy). Then, 4 μm sections were cut from paraffin blocks and stained with hematoxylin–eosin. Anti‐active caspase 3 antibody (Abcam) was used to reveal apoptotic cells by IHC.

### Statistical analysis

Data were analyzed using Student's *t*‐test and Fisher's exact test. Survival was estimated with Kaplan–Meier estimators and differences tested with the log rank test.

## Author contributions

Marco Giorgio planned and performed the experiments, analyzed the results, and wrote the manuscript. Massimo Stendardo performed the experiments. Enrica Migliaccio analyzed the results. Pier Giuseppe Pelicci planned the experiments and wrote the manuscript.

## Funding

No funding information provided.

## Conflict of interest

None declared.

## Supporting information


**Fig. S1** Representative FISH images of sections of different organs from G0, G3 and G5 TERC^−/−^P66SHC^+/+^ and TERC^−/−^p66SHC^−/−^ mice, stained with the Cy3 conjugated TelC PNA probe (grayscale panels), the colored panels show Cy3 and DAPI fluorescence pattern in a stained section of WT liver as control.
**Fig. S2** IHC analysis of testis slices from G3 and G5 TERC^−/−^ p66SHC^+/+^ and TERC^−/−^ p66SHC^−/−^ mice with anti‐activated caspase 3 antibody.
**Fig. S3** HE stained sections of quadriceps from G0 and G3 TERC^−/−^p66SHC^+/+^ or p66SHC^−/−^ mice.
