# Supplementary material for: P66SHC deletion improves fertility and progeric phenotype of late‐generation TERC‐deficient mice but not their short lifespan
Source: Aging Cell. 2016 Mar 10;15(3):446–54. doi: 10.1111/acel.12448 (PMC4854904; doi:10.1111/acel.12448)

**Supplementary Figure 1.** Representative FISH images of sections of different organs from G0, G3 and G5 TERC-/-P66SHC+/+ and TERC-/-p66SHC-/- mice, stained with the Cy3 conjugated TelC PNA probe (grayscale panels), the colored panels show Cy3 and DAPI fluorescence pattern in a stained section of WT liver as control.

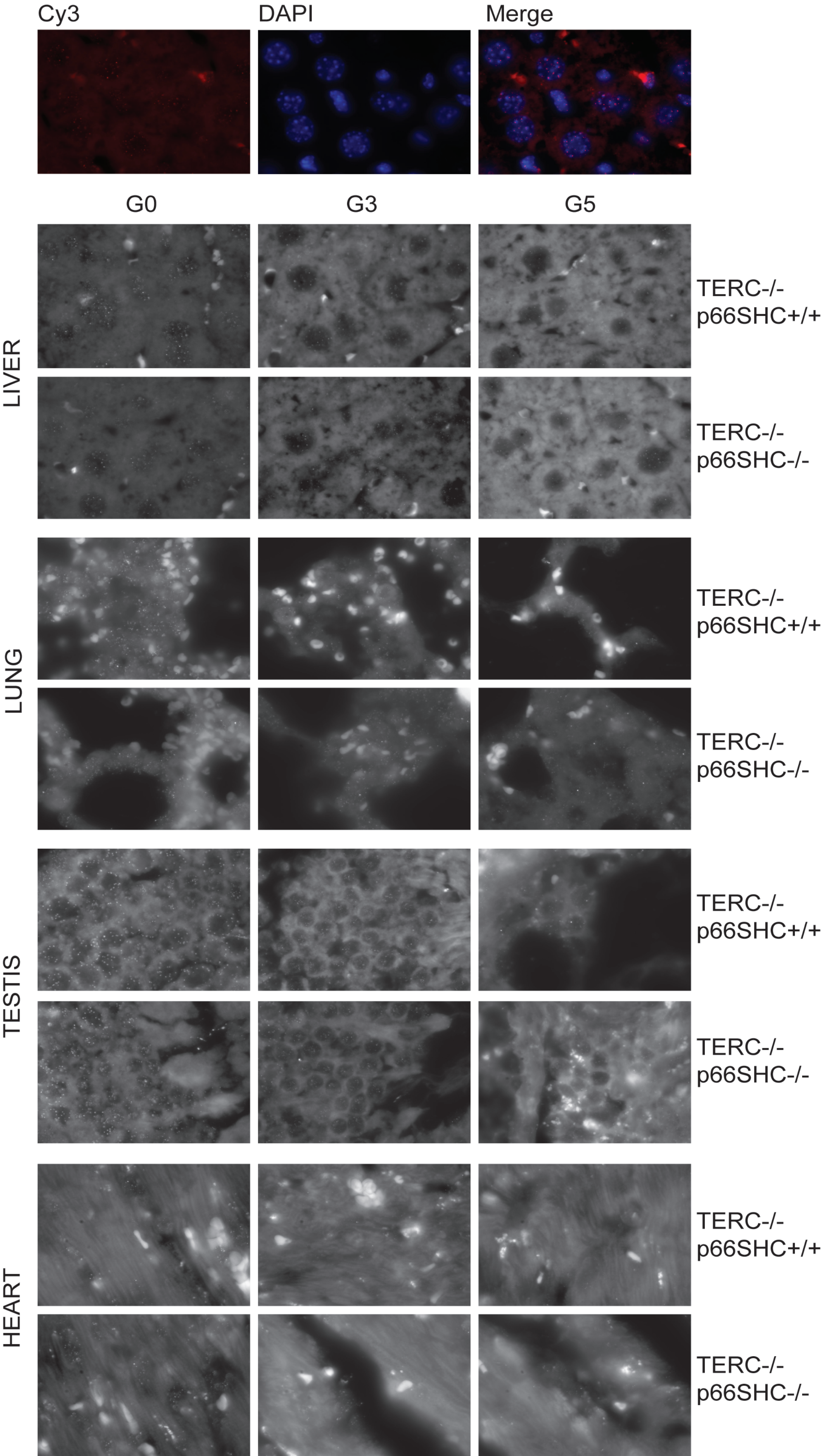

**Supplementary Figure 2.** IHC analysis of testis slices from G3 and G5 TERC-/- p66SHC+/+ and TERC-/- p66SHC-/- mice with anti-activated caspase 3 antibody. The arrows indicate positive cells.

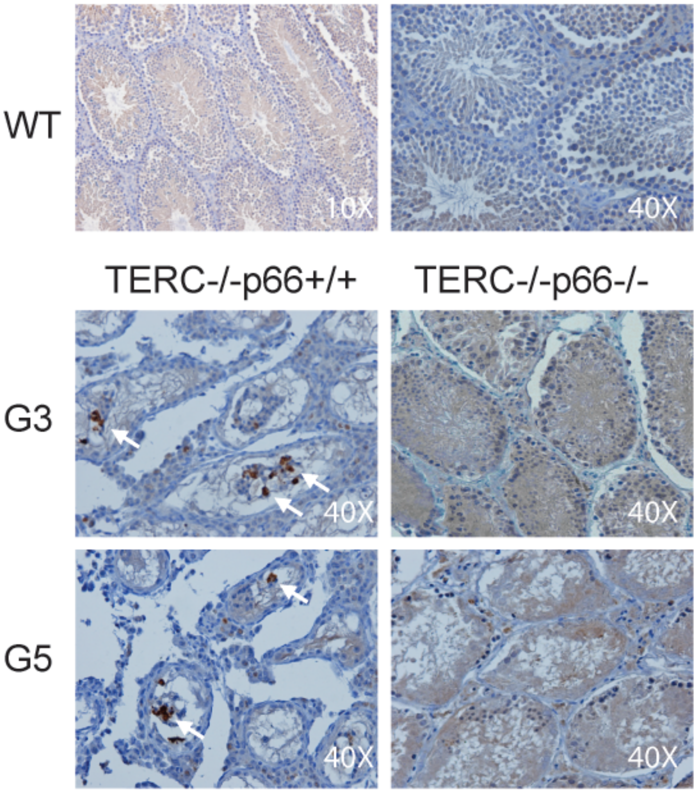


**Supplementary Figure 3.** HE stained sections of quadriceps from G0 and G3 TERC-/-p66SHC+/+ or p66SHC-/- mice

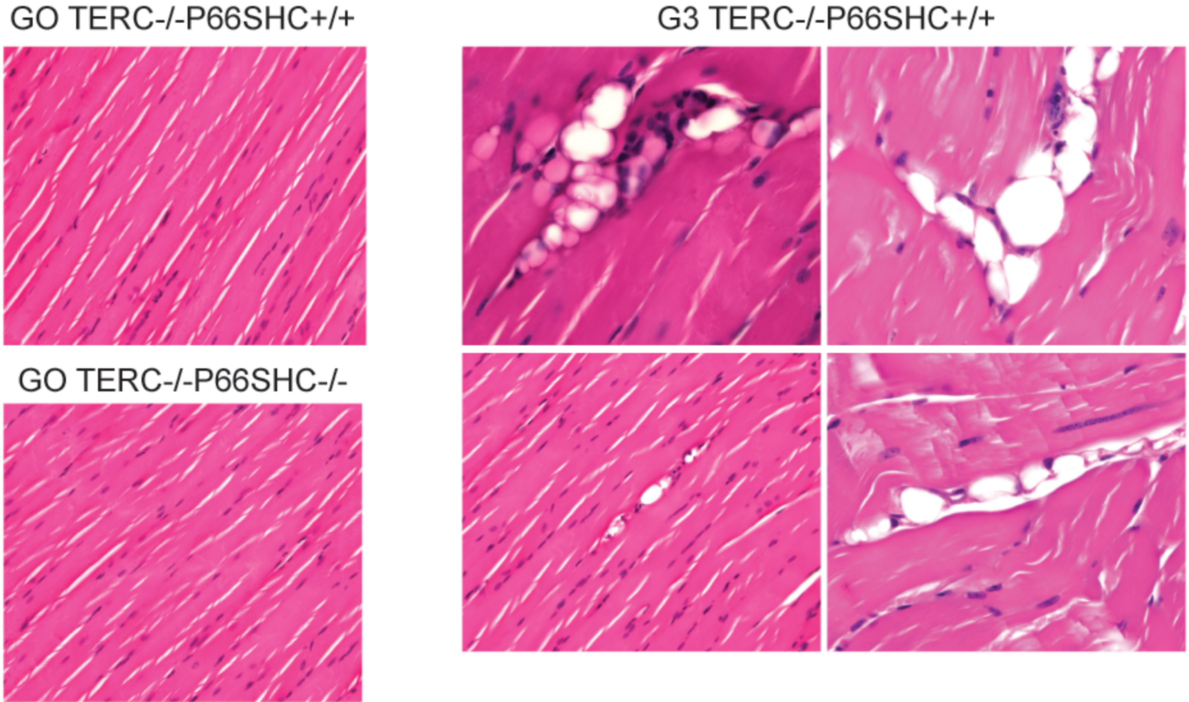

Supplement: Supplementary file 1 — Fig. S1 Representative FISH images of sections of different organs from G0, G3 and G5 TERC−/−P66SHC+/+ and TERC−/−p66SHC−/− mice, stained with the Cy3 conjugated TelC PNA probe (grayscale panels), the colored panels show Cy3 and DAPI fluorescence pattern in a stained section of WT liver as control. Fig. S2 IHC analysis of testis slices from G3 and G5 TERC−/− p66SHC+/+ and TERC−/− p66SHC−/− mice with anti‐activated caspase 3 antibody. Fig. S3 HE stained sections of quadriceps from G0 and G3 TERC−/−p66SHC+/+ or p66SHC−/− mice. [file ACEL-15-446-s001.pdf]
